# Miscellaneous Intelligence

**Published:** 1823-12-01

**Authors:** 


### MISCELLANEOUS INTELLIGENCE.

### Dr. MERRIMAN to Dr. JOHNSON.

Dear Sir,

I observe in the last number of the Medico-Chirurgical Review, an opinion respecting the vitality of infants, born at seven and eight months of utero-gestation, the accuracy of which appears very doubtful. The opinion is thus expressed in the review of Paris and Fonblanque on Medical Jurisprudence," page 140:—"Our authors ridicule as a conceit the idea entertained by Hippocrates, and even by many modern writers, that an infant could live at seven months, but rarely or never at eight. Whatever may be the degree of conceit in the business, *we believe it is a fact, that more children are born and live at seven, than at eight months of utero-gestation.*"

That this belief was once very general is readily admitted; but I had presumed that, among medical men, the opinion of the greater vitality of seven, than of eight months children, had been abandoned since the time of Mauriceau; who, in order to ascertain the fact, had numerous observations made, by Madame de la Marche, the celebrated midwife of the Hotel Dieu, at Paris, the result of which was a conviction on the part of Mauriceau, that "*more than one half of those who are born at*

*eight months will live, while of those born at seven months very few survive.*"

As an erroneous persuasion on this subject must necessarily, on some occasions, essentially influence our practice, it is of importance, should any facts be known, which invalidate Mauriceau's testimony, that they should be made public; but I am very sceptical as to the existence of any such facts. The experience which I have had, goes to confirm Mauriceau's statement. I have now lying before me, a list of premature births, in which the period of utero-gestation is distinctly marked. The list amounts to thirty-six cases of eight months children, and thirty-four of seven months. Of the *thirty-six* eight months children, there died during the month of child-bed only *eight;* but among the *thirty-four* seven months children, there died within the month—*twenty-one.*

It may be proper to remark, that the above are, all, cases of single births; but the observation holds good, and nearly in the same proportion, in twin cases. I observe, likewise, that of the children *still-born*, a much greater proportion were at seven, than at eight months of utero-gestation. As I know how desirous you are of establishing facts, I feel assured that you will excuse me for troubling you with this statement.

I remain, dear Sir, your's faithfully,

SAMUEL MERRIMAN.

34, *Brook Street, Grosvenor Square,*
*September* 13*th*, 1823.

### ULCERATION OF THE CÆCUM.

At page 470 of our last number we gave some particulars of a curious case of ulceration of the cæcum communicating with an external opening in an abscess of the groin. The case was transmitted to us by an intimate friend, the brother of the patient, and without the names of any of the parties concerned. Supposing the particulars had been furnished by one of the medical attendants, we published it as it came to hand; adding, however, the query at the end. We have since been sorry to learn that the *history* of the case was not correctly stated in some respects, and that, in consequence of this, and the mode of wording the case, the surgeon in charge imagined there was an insinuation thrown out against the fidelity of the minutes of dissection. We have only to declare that *we* never entertained such a suspicion, or threw out such an insinuation. If the case can bear such a construction, we confess ourselves incapable of perceiving where it lies. In order, however, that our readers may judge, we here give the history and dissection in the words of the surgeon himself.

" Mr. S. consulted me in the beginning of May 1823, concerning a tumor in the upper part of the right groin, which appeared to be formed by a mass of enlarged lymphatic glands, situated within and above the middle of Poupart's ligament. It soon became evident that suppuration had begun to take place, and the tumour was fomented and poulticed. On Sunday morning, June 1st, the patient sent to me a message that the abscess had burst, and that he was desirous that I should visit him at his own lodging. I found that the abscess was discharging an offensive discoloured pus through a small opening. Conceiving it to be desirable that the matter should have a free exit, I introduced a small director through the ulcerated opening and under the skin, and made the opening somewhat larger by cutting on the director with a lancet. This was followed by a further discharge of offensive matter. In the evening of this day Mr. S. was so well that although I had requested of him to remain in bed, I found him sitting up in his drawing room. On the following day, June 2d, he continued well, and was desirous of going into the country, to which, however, finding that the discharge was discoloured, and had a feculent odour, I did not consent. On the 3d of June, he observed, after taking a draught of decoction of bark, that that liquid escaped at the orifice in the groin, which stained the linen with the colour of the medicine. This seemed to excite in his mind a great degree of anxiety ; and from this time there was a good deal of disturbance of the general system. The pulse became frequent, the tongue was slightly furred, and he was very restless. It was observed that liquids taken into the stomach soon passed out at the external opening of the abscess, blended with feculent matter; nevertheless, solid feculent evacuations continue to take place daily in the usual manner. On Sunday, June 8th, the constitutional symptoms became aggravated. The pulse was more rapid, and the tongue was more furred. On Monday, June 9th, he was still worse, and on the following morning he began to sink, and expired about one, P. M.

"*Appearances on Dissection.*—In the lower part of the abdomen, immediately above the middle of Poupart's ligament, there was a cluster of enlarged lymphatic glands connected with the abscess. The ulceration which had taken place in the formation of the abscess had extended into the cæcum, in consequence of which there was a large opening forming a communication between the cæcum and the external wound.*

"The inner membrane of the intestine in the neighbourhood of the ulceration, to a considerable extent, was in a state of inflammation ; being of a very dark colour, in consequence of the vessels being loaded with blood. There were no other appearances of disease in the absomen.

"In the thorax the lungs were found unusually turgid with blood; and the surface of the right lung towards the posterior part was covered by a thin layer of coagulated lymph. In the eavity of the right pleura there were about six ounces of serous fluid."

It will be seen that the defects or omissions in the case, as originally published, are—First, It was *not* stated that the tumour had the appearance of being formed (as it turned out to be) of a mass of enlarged lymphatic glands in the groin. Secondly, it was mentioned that the tumour was *opened*—whereas, the abscess *burst spontaneously*, and the opening was merely enlarged superficially afterwards.

When we queried, whether there might not have been originally a hernia? we alluded to the hurt formerly received when jumping off a coach at Edinburgh, and never entertained, for an instant, the idea of there being a hernia when the abscess was opened. We conceived it possible, that had there been a partial hernia in the beginning, there might have remained some adhesion of the gut in the neighbourhood, to account for its partaking in the ulceration when the glands became suppurated. This was the idea that floated in our mind, and we never had the most remote intention of attributing blame to any person concerned. Neither did we ever entertain a doubt of the fidelity of the minutes of dissection. Since the publication of the case, we have seen a gentleman of talent and integrity, who was a witness of the examination, and vouches for its scrupulous correctness.

---

In the early part of January, 1824, will be published some Lithographical Plates of the Conformation of the Heart, by H. SEARLE.

---

" * Of course the mass of enlarged glands were adhering to the cæcum at their posterior surface."

*The following work came too late for our Bibliographical Record.*

(No. 28.) An engraved Representation of the Anatomy of the Human Ear, exhibiting in one view the external and internal parts of that Organ, in situ. Accompanied with a Plate of the Outlines and References, with copious Explanations. To which are added, Surgical Remarks on introducing the Probe and Catheter into the Eustachian Tube by the Nostril —on the Operation of puncturing the Membrana Tympani—and a synoptical Table of the Diseases of the Ear, their Classification, Seat, Symptoms, Cause, and Treatment. The whole designed as a Guide to Acoustic Surgery. By THOMAS BUCHANAN, C. M. Licentiate of the University of Glasgow, corresponding Member of the Phrenological Society of Edinburgh, and Surgeon to the Hull Dispensary for Diseases of the Eye and Ear. Folio, pp. 42, with plates. Price 12s. 6d. boards. London, 1823.

☞ *An account of this Work in our next number.*

---

A German translation of Dr. W. Philip's Work on Indigestion, by Dr. Hymans, has appeared at Rotterdam, in the present year.

Dr. Hasper, of Leipzig, has published a translation of the same work.

---

*Transactions of the Associated Apothecaries.* In our review of this work, it may be asked why we have not noticed the Introductory Essay occupying nearly 170 pages of the work. The plain answer is, that this Essay was printed *after* the rest of the work, of which we got an early sight, as a matter of favour; and thus the Introduction has been left unnoticed. We are free to confess, however, that we are not sorry this production of the sub-committee escaped us, for, in truth, we could not have said much in its favour.

---

### CLAPHAM RETREAT.

We have examined, with much attention, this excellent establishment, fitted up with great care and skill by Dr. Burrows. It is impossible not to admire the admirable accommodations, moral, medical, and domestic, which this institution affords to the unfortunate lunatic. Being superintended by a man of established reputation in medical science, it deserves, and we are confident it will soon obtain, the approbation and patronage of the profession.

---

### DR. SMITH'S LECTURES ON MEDICAL JURISPRUDENCE.

We are glad to find, by a prospectus which has just reached us, that Dr Gordon Smith, the ingenious author of a valuable work on forensic medicine, means to commence a course of lectures on the important subject of medical jurisprudence, "in which the prominent object will be to illustrate the application of the medical sciences to questions that come before the courts of justice concerning the human frame." If the utility of *writings* on political medicine be allowed—and it is now universally allowed—that of lectures must be still more useful, in consequence of the *illustrations* they afford of the various subjects discussed—and the experiments they admit of, which cannot be shewn in books.

We think such an undertaking must ensure the patronage of the profession, as soon as it is commenced and sufficiently known. It is Dr. G.'s intention to begin in the first week of January, and to give about 40 lectures in the course of three months. The time and place will be advertised hereafter. Meanwhile he requests that those who mean to favour the undertaking by their attendance will have the kindness to notify the same to the medical booksellers—which notification will be by no means binding on them, though it will enable the lecturer to make suitable arrangements for the occasion.

---

### DR. HARRISON TO THE EDITOR.

Dear Sir, *Holles Street, Cavendish Square, Nov.* 20*th,* 1823.

Having accidentally read, in your Review for September, some animadversions upon my picture, lately exhibited in Somerset House, I request the favor of you to publish the following statement, that your readers may be enabled to form a clear opinion upon the subject.

I had an opportunity to confer an act of civility upon Mr. Shee, which he desired to return, by presenting me with my portrait. Under these circumstances, and placing the greatest confidence in his professional skill and elegant accomplishments, I did not presume, first or last, to give an opinion, much less to interfere with any of the arrangements. The design, the execution, and even the idea of shewing the picture in Somerset House, were all Mr. Shee's;—he is therefore the gentleman to be referred to, for any supposed defect, in the plan, taste, or composition of the piece.

I have sent this letter for insertion in the next number of your Journal, anticipating your readiness to give it a place there, in order to maintain the impartiality of your Journal, and to perform an act of justice towards, dear Sir, Your obedient humble Servant,

EDWARD HARRISON.

☞ We consider the above explanation as perfectly satisfactory, and are happy to find that Dr. Harrison has exonerated himself completely from the charge of bad taste which was brought against the picture alluded to.—Editors.

---

### INJECTING MACHINE FOR THE STOMACH OR BOWELS.

For many months past we have been in the habit of employing Mr. Read's patent injecting apparatus, which is so small as to be carried in the waistcoat pocket, and yet so powerful as to throw fluids to a great distance. The object of our present notice, however, is to inform our readers that Mr. Read has adapted to the instrument a flexible elastic tube most admirably calculated for throwing liquids into the stomach, and then extracting them, in cases of poisoning. We have attentively examined the instrument, and we know it is approved of by Sir Astley Cooper, and some of the first surgeons in this metropolis. We think it of so much importance, that we seriously recommend it to every private practitioner.

It may be procured of Mr. Pepys, Instrument Maker, 22, Poultry; Mr. Stoddart, 401, Strand; and Mr. Thompson, Great Windmill Street, Haymarket.

---

We observe by the last list of the Royal College of Physicians that the name of Dr. Thornton has been struck off. We think Dr. Thornton's conduct, in aiding the Whitlaw imposition on public credulity, richly deserved this mark of disapprobation—as we observed several numbers back in this Journal. Sincerely do we hope that immoral as well as unprofessional conduct shall be often served in this way in future.

---

Nos. 11, 12, 13, and 14, of this Journal are entirely out of print. The back numbers will either be re-printed, or a new series commenced with the next volume. The latter is more probable, as very few remain of any of the back numbers from No. 1 to No. 10.

